# Preferences and Willingness to Pay for Herpes Zoster Vaccination Among Chinese Adults: Discrete Choice Experiment

**DOI:** 10.2196/51242

**Published:** 2024-08-09

**Authors:** Yiqi Xia, Ming Wang, Mingzheng Hu, Yanshang Wang, Beibei Yuan, Dawei Zhu, Ping He

**Affiliations:** 1 School of Public Health Peking University Beijing China; 2 China Center for Health Development Studies Peking University Beijing China; 3 Department of Pharmacy Administration and Clinical Pharmacy School of Pharmaceutical Sciences Peking University Beijing China; 4 International Research Center for Medicinal Administration Peking University Beijing China

**Keywords:** herpes zoster, vaccine preference, willingness to pay, discrete choice experiment, mixed logit model

## Abstract

**Background:**

The incidence of herpes zoster (HZ) is rapidly increasing, causing both clinical and economic burdens in China. Very little is known about Chinese residents’ HZ vaccine preferences and willingness to pay (WTP) for each vaccination attribute.

**Objective:**

This study aims to elicit the preferences of Chinese urban adults (aged 25 years or older) regarding HZ vaccination programs and to calculate WTP for each vaccination attribute.

**Methods:**

In this study, we interviewed 2864 residents in 9 cities in China. A discrete choice experiment was conducted to investigate the residents’ preferences for HZ vaccination and to predict the uptake rate for different vaccine scenarios. A mixed logit model was used to estimate the preferences and WTP for each attribute. Seven attributes with different levels were included in the experiment, and we divided the coefficients of other attributes by the coefficient of price to measure WTP.

**Results:**

Vaccine effectiveness, protection duration, risk of side effects, place of origin, and cost were proven to influence Chinese adults’ preferences for HZ vaccination. The effectiveness of the HZ vaccine was the attribute that had the most predominant impact on residents’ preferences, followed by protection duration. The residents were willing to pay CN ¥974 (US $145) to increase the vaccine effectiveness from 45% to 90%, and they would barely pay to exchange the vaccination schedule from 2 doses to 1 dose. It is suggested that the expected uptake could be promoted the most (by 20.84%) with an increase in the protection rate from 45% to 90%.

**Conclusions:**

Chinese urban adults made trade-offs between vaccine effectiveness, protection duration, place of origin, side effects, and cost of HZ vaccination. Vaccine effectiveness was the most important characteristic. The residents have the highest WTP (CN ¥974; US $145) for enhancing the effectiveness of vaccines. To maximize HZ vaccine uptake, health authorities should promote vaccine effectiveness.

## Introduction

Herpes zoster (HZ), or shingles, is caused by the reactivation of latent varicella-zoster virus infection, which typically manifests itself as a localized, painful dermatomal rash [1]. The most common complication for HZ is postherpetic neuralgia, which can cause severe and burning pain and can greatly limit daily activities [2,3]. The severity of HZ is a major public health issue worldwide, especially in China [4], imposing a substantial burden on both patients and the health care system [5]. The global incidence of HZ was 3 to 5 per 1000 person-years, which continued to rise by 2.5% to 5% every year [6,7], and was 2.9 to 5.8 per 1000 person-years in mainland China [5,8]. In the United States, the economic burden for HZ could exceed US $2 billion per year, resulting in more than 60,000 quality-adjusted life-years lost per year [9]. A recent study conducted in Shandong, China, assessed the disease burden for HZ as 59.99 disability-adjusted life-years per 100,000 population [10]. Data gathered from Yinzhou district, China, showed the inpatient and outpatient cost per new-onset HZ was approximately CN ¥8116.9 (US $1207) and CN ¥560.2 (US $83), respectively [11]

Existing evidence supports that vaccination is the best way to reduce the incidence of HZ [12,13]. In addition, a majority of studies found it to be cost-effective to vaccinate against HZ [14]. Currently, 2 types of HZ vaccines are commonly used globally, one of which is a recombinant zoster vaccine available in China since 2020 and recommended for adults older than 50 years [8,15]. However, HZ vaccine uptake remains relatively low worldwide [15], along with the low willingness to pay (WTP) for HZ vaccines [8]. Vaccine decisions are often determined by an individual’s requirements and interests [16]. Whether to get vaccinated is, hence, based on internal and vaccine-specific characteristics, such as personal values [17], vaccine effectiveness [18], and cost [19], which can be interpreted as vaccine preferences [19]. Consequently, it is vital to consider the public’s preferences for HZ vaccination.

Studies investigating the public’s preferences for HZ vaccination are limited and fragmented. Although a growing number of studies have tried to find the main barriers to HZ vaccine acceptance [20], only 3 published studies have examined HZ vaccine preferences among older people in the context of high- and low-income countries with discrete choice experiments (DCEs): 2 from high-income countries [21,22] and 1 from low-income countries [23]. Zhang et al [23] investigated the HZ vaccine preferences among older Chinese residents with a relatively small sample size (176 residents); but still, very little is known about HZ vaccine preferences among adults in low-income countries. There is no comprehensive, nationwide survey about HZ vaccine preferences and WTP for different HZ vaccine attributes of the Chinese population across regions, although China is one of the prominent candidates in the global contest for vaccine research and development [24]. Moreover, it is worth studying adults younger than 50 years because the younger population may affect HZ vaccination by influencing their parents who are probably older than 50 years, and the study may provide an opportunity to compare the differences in preferences for HZ vaccination between younger and older populations [25].

In this study, we aimed to use a DCE to explore the preferences for different attributes of HZ vaccination among the Chinese urban population aged 25 years and older and to evaluate the residents’ WTP for each attribute. We further calculated the probability of selecting different HZ vaccines to better describe the preference characteristics.

## Methods

### DCE Study Design

#### Overview

DCE, originating from mathematical psychology, is widely used to capture preferences in vaccination since the evidence showed that DCEs yielded accurate predictions of actual choices [19,26,27]. When measuring vaccine preferences with DCEs, individuals are given a series of alternative vaccine scenarios and are asked to select their preferred scenario [19,28]. Within each scenario, the vaccines are described by their attributes (eg, effectiveness) with variants of corresponding levels (eg, the effectiveness of 50% vs 90%) [28]. By assessing individual’s responses to different combinations of attribute levels, preferences for vaccine attributes are able to be obtained and described [29,30].

Here, we developed a cross-sectional DCE survey following standard steps [31,32] and administrated the experiments in July 2022 among adults aged 25 years and older in 9 cities in China. The survey consisted of DCE questions and measures of respondents’ characteristics (sex, age, educational level, marriage status, occupation, and annual net household income in 2021). Questionnaires were conducted face-to-face by well-trained and eligible investigators (mainly medical school students).

#### Attributes and Levels

The selection of vaccine attributes and their levels is key for ensuring the validity of DCEs [33]. Since there are limited studies about Chinese residents’ HZ vaccine preferences, we selected 7 relevant HZ vaccination attributes and their levels based on literature about HZ vaccine preferences in China and other countries and Chinese residents’ preferences for another vaccine [21-23,28,34]. We also referred to existing HZ vaccine marketing information and conducted a pilot study to ensure that the parameters in these references were applicable to conditions in China: (1) effectiveness (eg, 45%); (2) protection duration (eg, 2 years); (3) number of vaccine doses (eg, 1 dose); (4) probability of influenza-like symptoms (eg, 1/100); (5) probability of skin reaction (eg, 5/100); (6) place of origin (eg, imported); and (7) price (eg, CN ¥400 [US $59]). The details of the attributes and levels are displayed in Table 1.

**Table 1:** Attributes and levels for the discrete choice experiment.

Attribute	Levels for experimentally designed vaccines
Effectiveness (%)^a^	45^b^, 60, 75, and 90
Protection duration (years)^c^	2^b^, 5, 10, and 20
Number of vaccine doses^d^	1 dose^b^ and 2 doses (2 months apart)
Probability of influenza-like symptoms^e^	1/100^b^, 5/100, 10/100, and 20/100
Probability of skin reaction^f^	1/100^b^, 5/100, 10/100, and 20/100
Place of origin	Imported and domestic^b^
Price (CN ¥)	0^b^, 400 (US $59), 800 (US $119), and 1200 (US $178)

^a^Effectiveness is the degree to which the prevalence of the disease decreases after vaccination, which can also be interpreted as protection rate. Suppose there are 1000 people, and with everyone unvaccinated, 33 people might have herpes zoster (HZ) in the next 5 years. If everyone is vaccinated against HZ with a 45% vaccine effectiveness, the number of people who will have HZ in the next 5 years will drop to 18, and so on.

^b^Denotes the reference level.

^c^Protection duration refers to the duration of immune protection against the infectious disease targeted after vaccination.

^d^The number of doses refers to the total number of doses required to complete the vaccination process.

^e^Influenza-like symptoms refer to fever, fatigue, muscle pain, headache, and nausea that may occur in recipients after vaccination. With a 1/100 probability, 1 in 100 recipients may suffer from 1 or more of these symptoms; with a 20/100 probability, 20 in 100 recipients may suffer from 1 or more of these symptoms.

^f^Skin reactions refer to symptoms such as redness, swelling, hard nodules, pain, and itching at the vaccination site that may occur when the recipient is vaccinated. With a 1/100 probability, 1 out of 100 recipients may suffer from 1 or more of these symptoms; with a 20/100 probability, 20 out of 100 recipients may suffer from 1 or more of these symptoms.

#### Experiment and Questionnaire Design

The vaccine scenarios in each alternative were determined by an experimental design using the attributes and levels in Table 1. Combining the 7 attributes with each level results in 4096 (4×4×2×4×4×2×4) hypothetical HZ vaccination alternatives. Since it is not feasible for a single respondent to answer all these 4096 alternatives, we used an orthogonal experimental design to generate a sample of alternatives from all these 4096 alternatives [35,36]. Since orthogonal designs are more intuitive and require fewer choice sets and thus shorter questionnaires than efficient designs with the same number of participants, an orthogonal design was used to counterbalance a more precise estimation of the coefficients and fewer choice sets [28,37]. A total of 32 choice questions were shown to sufficiently estimate all the main attributes. We used a blocked design [38] to further divide 32 choice tasks into 4 versions of questionnaires containing 8 choice sets each, which avoided a lower response rate or lower response reliability caused by presenting a single individual with a large number of choice sets. Each choice set presented 2 alternative vaccines in terms of 7 attributes and an option of no HZ vaccination (Table 2), a full version of the questionnaires is presented in Multimedia Appendix 1. To specify the attributes, each questionnaire started with a detailed description of the attributes and their levels. Additionally, the well-trained investigators were told to explain the meaning of each attribute and to ensure the comprehension of the respondents before filling in the questionnaire. During the filling process, the respondents could ask questions about the attributes at any time.

**Table 2:** An example of choice sets^a^. The description of the attributes and their levels are as follows: “Thank you for agreeing to take part in this survey. Assuming that you have two options for herpes zoster vaccine that differ in price, effectiveness, protection duration, adverse effects, etc, please choose the vaccination option you prefer based on your personal preference among the pairs of options below. When you make a choice, please assume that all conditions are the same for vaccine 1 and vaccine 2 except for the factors listed.” Towards the end of the questionnaire, the respondent was given the following choice: “Which do you prefer: vaccine 1; vaccine 2; neither.”

Attribute	Vaccine 1	Vaccine 2
Effectiveness (%)	90	45
Protection duration (years)	2	5
Number of vaccine doses	2 doses (2 months apart)	1 dose
Probability of influenza-like symptoms	1/100	5/100
Probability of skin reaction	20/100	1/100
Place of origin	Imported	domestic
Price (CN ¥)	0	400 (US $59)

^a^There are 4 versions of the discrete choice experiment questions, each with 8 questions, and each respondent is randomly assigned a version of the questions.

#### WTP for the Attributes

In total, 7 types of attributes were identified to be potentially important to HZ vaccination choices, including the price of the vaccine that allows us to generate estimates of WTP for each vaccine attribute [39]. In other words, we could obtain the individual’s WTP for desirable attributes and analyze the effect of changing attribute levels on WTP indirectly with the price attribute in the DCE questionnaire through the ratios between the coefficients of the price (cost) attribute with the other HZ vaccination attributes [30]. For example, the WTP for switching the protection duration to 5 years from the reference level (2 years) equaled the coefficient of the variable “duration 5 years” divided by the coefficient of the variable “price.” The levels of the cost attribute may inevitably impact the WTP estimates though, so we tried to avoid this problem by setting the levels rigorously according to the literature [40].

### Study Population

#### Survey Sampling

A 2-stage random sampling method was adopted. In the first stage, 9 cities were selected according to gross domestic product per capita and the number of permanent urban residents (except Tibet and Xinjiang) using the probability-to-size sampling method, including 5 cities in eastern China (Beijing, Shenzhen, Weifang, Shaoxing, and Changchun), 2 cities in central China (Wuhan and Zhengzhou), and 2 cities in western China (Nanning and Zunyi). In the second stage, high and low economic level districts (county) were selected in each city, respectively, and 2 community health service centers or township health centers were then selected in each district (county).

We recruited respondents randomly with acknowledgments after arriving at the health service centers. Eligible respondents were aged 25 years and older, living in China, without cognitive impairments, and able to read and understand Chinese [21,33]. A pilot study was conducted among 300 residents in Beijing in July 2021 to examine whether the survey was acceptable, well-understood, and valid.

#### Sample Size

Initially, we calculated the minimum sample size according to a rule of thumb suggested by Jonson and Orme [41] and found a sample size of 83 in each district (county) would be desirable for the main effects model. As a result, we decided to choose 300 individuals in each city, which exceeded the desirable sample size and comprised a sample size of at least 2700 randomly selected residents in 9 cities. We guaranteed the respondents’ voluntary participation and privacy, and verbal consent of the respondents was obtained. The investigators gave a detailed explanatory statement to each respondent about the study.

### Statistical Analysis

The DCE results were estimated by taking each choice among the 3 options (“two HPV vaccination” alternatives and a “no HPV vaccination” alternative) [28]. A mixed logit model was used to analyze DCE data [42]. We checked the convergence of the model with all parameters randomly distributed, 500 draws used, and seed set to 12,345 for reproducibility. The utility equation can be expressed as follows:

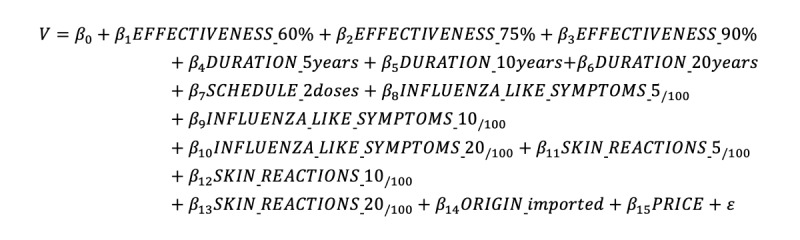



where *V* is the utility from each choice. *β*_0_ is a constant reflecting respondents’ preferences for receiving HZ vaccination relative to “no HPV vaccination.” *β*_1_-*β*_15_ are coefficients of the attributes indicating the weights relative to their reference levels, where larger values of *β* indicate greater utility and more preferred attributes. *ε* is a random error term. We regulated the statistical significance of a coefficient as *P*≤.05, and we expected all attributes to be statistically significant. The sign of a coefficient reflects whether the attribute has a positive or negative effect on vaccine preferences. In our assumption, the estimated attribute levels of “SCHEDULE” and “INFLUENZA_LIKE_SYMPTOMS” and the attribute “PRICE” would have a negative sign, and the signs of other coefficients would be positive. The price attribute was treated as a continuous variable, while other attributes were dummy-coded [43]. We also explored the preferences for HZ vaccination between different age, sex, and income.

To investigate the WTP for each attribute, the monetary value for other attributes was calculated by the ratios between the coefficients of out-of-pocket cost (price of the vaccine) with the other HZ vaccination attributes [34]. It can be interpreted as a tradeoff in monetary value to achieve an increase or a decrease in 1 level of an attribute. For instance, *β*_1_/*β*_15_ indicates how much the respondents were willing to pay for a vaccination with a 60% protection rate instead of the reference level of protection rate (45%), holding all other attributes unchanged. CIs of the WTP were calculated by bootstrapping [44].

We further calculated the probability of individuals choosing a vaccination with specified attributes to convey understandable information to policy makers. The probability of choosing a vaccination program is defined as:

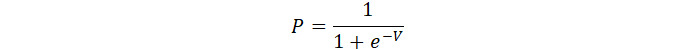



where *V* is defined as in Equation 1. The base case of the HZ vaccination program was a 45% protection rate, protection duration of 2 years, vaccination schedule of 1 dose, 1/100 risk of influenza-like symptoms, 1/100 risk of skin reaction, and originated from domestic place, which was the combination of the reference level of each attribute (except for cost attribute). We presented these results in a “tornado” graph to show the marginal effect of changing each attribute at a time relative to the base case, holding all other attributes constant [28,34]. All heterogeneity were considered in the calculation of the mean uptake. We performed all statistical analyses in STATA (version 15.0; Stata Corp).

### Ethical Considerations

The respondents provided informed consent before filling in the questionnaire and agreed to the use and publication of their data in journal papers. The questionnaire was completely anonymous, and the data were protected by privacy law. During the process of filling in the questionnaire, all respondents could withdraw from the survey at any time. This study was approved by the Peking University institutional review board (IRB00001052-20062). No financial compensation was offered to the participants. All procedures performed involving human respondents were in accordance with the ethical standards and with the 1964 Declaration of Helsinki.

## Results

### Respondents

After the pilot study, we confirmed the acceptability and validity of the survey, and mild changes were made to the description texts of the attributes in the questionnaire. Table 3 presents the respondents’ characteristics. A total of 2864 urban residents from 9 cities consented and participated in the survey. Among them, 1523 (53.18%) urban residents were from the eastern region, 641 (22.38%) urban residents were from central China, and 700 (24.44%) urban residents were from the western region. The mean (SD) age of the respondents was 48.14 (16.55) years, ranging from 25 to 95 years. Most respondents were female (1746/2864, 60.96%), married (2463/2864, 86%), and at least had a bachelor’s degree (706/2864, 24.65%). Annual net household income in 2021 was evenly distributed and the majority of the respondents were manual labor (1053/2864, 36.77%).

**Table 3:** Sociodemographic characteristics of the study respondents.

Characteristics		Frequency, n (%)
**City**		
	Eastern region	1523 (53.18)
	Central region	641 (22.38)
	Western region	700 (24.44)
**Sex**		
	Female	1746 (60.96)
	Male	1118 (39.04)
**Age group (years)**		
	25-39	1114 (38.90)
	40-59	929 (32.44)
	≥60	821 (28.66)
**Marital status**		
	Unmarried	252 (8.8)
	Married	2463 (86)
	Divorced or widowed	149 (5.2)
**Education**		
	Primary and below	537 (18.75)
	Middle school	588 (20.53)
	High school	581 (20.29)
	Junior college	452 (15.78)
	Bachelor’s degree and higher	706 (24.65)
**Occupation**		
	Manual laborer	1053 (36.77)
	Retiree	613 (21.4)
	Unemployed	463 (16.17)
	White collar and professional	735 (25.66)
**Annual net household income in 2021**		
	CN ¥40,000 (US $5,947) and below	741 (25.87)
	CN ¥40,000 (US $5,947) to CN ¥80,000 (US $11,894)	641 (22.38)
	CN ¥80, 000 (US $11,894) to CN ¥120,000 (US $17,841)	644 (22.49)
	CN ¥120,000 (US $17,841) and more	838 (29.26)

### DCE Results

Table 4 shows the regression results of residents’ preferences for HZ vaccination. The results indicated that all attributes had an effect on residents’ preferences for HZ vaccination (all *P*<.01), except for vaccination schedules. The coefficient of vaccination (constant) was significantly positive (*P*<.001), representing that on average the residents were more likely to vaccinate against HZ regardless of the attributes and levels described in the vaccine profile. It is suggested that the higher the protection rate, the longer the protection duration, the lower the risk of flu-like or dermal symptoms, and the lower the cost—the more likely that the HZ vaccination would be preferred. The positive or negative signs were consistent with our prior hypotheses except that respondents preferred the domestic HZ vaccine rather than the imported one. The positive sign given to the coefficients protection rate and protection duration indicated that respondents preferred an HZ vaccination generating a higher degree of protection and a longer protection duration over an HZ vaccination with lower effectiveness and fewer years of protection. The negative signs for influenza-like symptoms and skin reactions indicated that respondents preferred HZ vaccines with a lower probability of side effects. The attribute with the greatest magnitude of association with HZ vaccine preference was effectiveness, followed by protection duration.

**Table 4:** Preference of attributes of herpes zoster vaccines^a^.

Attributes		Coefficient (SE)		95% CI		*P* values
Constant (vaccination)		2.804 (0.152)		2.506 to 3.102		<.001
**Protection rate (%; reference level: 45%)^b^**						<.001
	60	0.958 (0.450)	0.870 to 1.046			
	75	1.751 (0.577)	1.638 to 1.864			
	90	2.746 (0.746)	2.600 to 2.892			
**Protection duration (years; reference level: 2 years)**						<.001
	5	0.404 (0.446)	0.317 to 0.491			
	10	0.678 (0.495)	0.581 to 0.775			
	20	1.013 (0.500)	0.915 to 1.111			
**Number of vaccine doses (reference level: 1 dose)**						
	2 doses	–0.001 (0.300)	–0.059 to 0.058		.98	
**Probability of influenza-like symptoms (reference level: 1/100)**						
	5/100	–0.162 (0.045)	–0.249 to –0.074		<.001	
	10/100	–0.166 (0.550)	–0.274 to –0.058		<.001	
	20/100	–0.272 (0.046)	–0.362 to –0.183		.002	
**Probability of skin reaction** **(reference level: 1/100)**						<.001
	5/100	–0.154 (0.042)	–0.236 to –0.072			
	10/100	–0.233 (0.049)	–0.329 to –0.136			
	20/100	–0.291 (0.046)	–0.382 to –0.201			
**Place of origin** **(reference level: domestic)**						<.001
	Imported	–0.354 (0.037)	–0.427 to –0.282			
	Cost	–0.003 (0.000)	–0.003 to –0.002			

^a^Normal distribution for random coefficients used on all attributes.

^b^The attribute “effectiveness” was coded as “protection rate.”

We presented subgroup analysis for different sex, age, and net household income in 2021 in Multimedia Appendix 2. The trends and signs of preferences for various attributes of the HZ vaccines were consistent across different sex, age groups, and net household income levels. It was illustrated that the male individuals cared more about side effects than the female individuals. The younger and older residents were more sensitive to most attributes than the middle-aged residents. The richer the residents were, the more they were concerned about the effectiveness and protection duration of the HZ vaccination, while the residents whose net household income in 2021 was less than CN ¥80,000 (US $11,894) preferred domestic vaccines more than residents with income more than CN ¥80,000.

### WTP for the Attributes

Based on Table 4, respondents showed their WTP to achieve an improvement in 1 level of an HZ vaccination attribute in Table 5. When the vaccine protection rate increased from 45% to 60%, 75%, and 90%, the respondents’ WTP for HZ vaccine increased by CN ¥340 (US $51), CN ¥621 (US $92), and CN ¥974 (US $145), respectively. The longer the protection duration, the higher the residents were willing to pay for HZ vaccine. WTP for domestic vaccines instead of imported vaccines was CN ¥126 (US $19). The residents were willing to pay CN ¥103 (US $15) to get vaccination with a 1/100 risk of skin reaction instead of 20/100 risk of skin reaction. The higher the incidence of influenza-like symptoms, the less the residents were willing to pay for HZ vaccine. Compared with 2-dose vaccination schedules, residents were more willing to pay for 1-dose HZ vaccine.

**Table 5:** Residents’ willingness to pay for different attributes of a vaccination program.

Attributes		Monetary value (CN ¥)	
		Mean	95% CI
**Protection rate (%; reference level: 45%)**			
	60	339.843 (US $50.526)	304.702 to 374.983
	75	620.937 (US $92.318)	572.224 to 669.650
	90	973.724 (US $144.768)	904.968 to 1042.479
**Protection duration (years; (reference level: 2 years)**			
	5	143.238 (US $21.296)	111.432 to 175.043
	10	240.526 (US $35.760)	204.035 to 277.016
	20	359.236 (US $53.409)	320.115 to 398.357
**Number of vaccine doses (reference level: 1 dose)**			
	2 doses	–0.257 (–US $0.038)	–21.092 to 20.579
**Probability of influenza-like symptoms (reference level: 1/100)**			
	5/100	–57.323 (–US $8.522)	–88.532 to –26.113
	10/100	–59.001 (–US $8.772)	–97.397 to –20.604
	20/100	–96.595 (–US $14.361)	–128.627 to –64.563
**Probability of skin reaction** **(reference level: 1/100)**			
	5/100	–54.534 (–US $8.108)	–83.970 to –25.098
	10/100	–82.658 (–US $12.289)	–117.404 to –47.912
	20/100	–103.322 (–US $15.361)	–135.743 to –70.902
**Place of origin** **(reference level: domestic)**			
	Imported	–125.687 (–US $18.686)	–152.053 to –99.320

### Predicted Uptake Rates of HZ Vaccination

The cost attribute was treated as a continuous variable and was mainly used to calculate the WTP. According to the literature, we dropped the cost attribute from this analysis to show more precise uptake rates of HZ vaccines to avoid confusion about the hypothetical cost levels because levels of the cost attribute may not represent the real price of current or future HZ vaccination programs [28]. The base case is indicated as zero change in the probability of the x-axis and the data presented a change in uptake from the base case (Multimedia Appendix 3). The predicted uptake could be promoted by 20.84%, 14.46%, and 7.72% if the protection rate was 90%, 75%, or 60% instead of a protection rate of 45% in the base case, respectively, holding all other attributes constant. Changes in other attributes and levels also had a relatively large impact on the expected uptake rate, especially when increasing the protection duration to 20 or 10 years from 2 years in the base case or substituting the imported vaccines in the base case with domestic ones. The vaccination schedule could barely interfere with the uptake rate.

## Discussion

### Principal Findings

Despite the increasing incidence of HZ and its clinical and economic burden on both patients and society, there was no evidence to indicate Chinese residents’ HZ vaccine preferences for promoting the vaccination uptake rate. In this DCE study of 2864 residents on preferences for HZ vaccination in 9 cities in China, we found that preferences among adults aged 25 years and older were affected by vaccination characteristics. Specifically, residents considered HZ vaccine effectiveness to be the most essential attribute, followed by protection duration. Cost had the mildest impact on residents’ preferences, and the number of doses did not seem to affect the preferences for HZ vaccination. We further explored how residents made a trade-off between monetary value and vaccine characteristics. Finally, we declared explicit results about the probability of choosing different HZ vaccines.

Vaccine effectiveness, protection duration, probability of influenza-like symptoms, probability of skin reactions, place of origin, and cost all showed to influence the residents’ preferences for HZ vaccination. The residents preferred to select the HZ vaccine with a higher protection rate, longer protection duration, fewer side effects, and lower prices. This has been confirmed in several previous studies about preferences for HZ vaccination in the United States [21] and China [23] and other vaccines’ acceptance [28,34,45]. The positive or negative signs of most of the coefficients in this study were consistent with our prior hypotheses and thus, showed theoretical validity [28]. Our study unexpectedly drew the same conclusions as a recent study about influenza vaccine preferences [43] that Chinese residents were more likely to select domestic vaccination. It is suggested that Chinese residents may have more confidence and trust in domestic vaccines nowadays, so the Chinese national immunization program should recognize the importance of developing domestic vaccines [46]. Respondents slightly preferred vaccination schedules of 1 dose over 2 doses, despite the coefficient was not significant. Similar studies about the HZ vaccine, hepatitis B vaccine, and COVID-19 vaccine all demonstrated that Chinese residents preferred shorter vaccination regimens [21,34,45], which could somehow prove the validity of our prior hypothesis.

We calculated the extent to which the residents preferred each attribute and level. Among all 7 attributes, effectiveness was the most important vaccine characteristic and protection duration was the next most important attribute. Specifically, increasing the protection rate from the lowest level to the highest level could yield 2.7 times as much utilities as increasing the same degree of protection duration of HZ vaccination. Although a recent study in the United States declared that preferences for HZ vaccination were influenced the most by total cost and then vaccine effectiveness [21], studies about German travelers choosing travel vaccines [47] and girls in the Netherlands choosing among HPV vaccinations [28] all showed that vaccine effectiveness was the most predominant attribute. The same results were derived from studies about preferences for COVID-19 vaccination [33] and influenza vaccination [43] in China. Zhang et al [23] found that the most concerning HZ vaccine attribute for Chinese residents was “vaccination of people surrounding them”; however, this attribute was not included in our study. After excluding attributes not presented in our study, vaccine efficacy remained the most significant attribute identified in most research [23,28,33,43,47]. In this study, place of origin, influenza-like symptoms, dermal reactions, and cost associated with HZ vaccination were of lesser importance, and the least important characteristic was the number of vaccine doses, which were highly consistent with another study about HZ vaccine preferences [21]. Consequently, promoting the effectiveness and protection duration while reducing side effects is a practical way to increase HZ vaccine uptake.

We found that the residents were willing to pay the most to alter the HZ vaccine from 45% effectiveness to 75% effectiveness and 90% effectiveness with CN ¥621 (US $96) and CN ¥974 (US $151), respectively, followed by spending CN ¥359 (US $56) to exchange HZ vaccine with 2 years protection duration to a protection duration of 20 years, again showing the importance of vaccine effectiveness and protection duration. Otherwise, the residents were willing to pay CN ¥103 (US $16) to get vaccination with a 1% risk of skin reaction instead of 20% risk of skin reaction. The trend of change in WTP was similar to the trend in previous studies [23,30,34], which reminded the policy makers to have an awareness of how to price the HZ vaccines with different attribute levels. Hypothetically, if there were an imported HZ vaccine of 45% effectiveness, 2 years of protection, and 20% probability of side effects (a combination of the reference levels), it may be acceptable to pay CN ¥1658 (US $257) more for domestic HZ vaccines of an effectiveness of 90%, 20 years of protection, and 1% probability of both influenza-like symptoms and skin reactions. An advantage of using DCE is to measure WTP indirectly rather than to obtain WTP by asking relevant questions directly [28,30]. Despite evidence that suggests that the inclusion and levels of the cost attribute could influence the estimates [40], we found no evidence proving that including a cost attribute could cause changes in vaccine preferences [48].

### Strengths and Limitations

Several limitations should be considered when interpreting our results. First, although we selected the most relevant attributes based on the literature, we were unable to cover all aspects of vaccination services. For instance, we did not include the location of vaccination as an estimated attribute as many studies did [21,34], but our investigation took place in community health centers with the respondents who lived nearby and would probably get vaccinated in their community, saving us from asking the place of vaccination. Second, this study surveyed residents in urban areas, which precludes the generalization of the findings [49]. Despite this, a direct policy implication is the improvement of vaccine effectiveness will increase the uptake of HZ vaccination in urban China. Third, our sample contained a relatively large number of eastern region, female, and married residents. Preference heterogeneity among Chinese residents requires future investigation.

There are also some notable strengths. To our knowledge, this is the first study to investigate the preferences for HZ vaccination comprehensively in China. Our findings verified that the uptake rate of the HZ vaccine can be explained by complex vaccination attributes, particularly vaccine effectiveness. We provide new information about WTP by evaluating the tradeoff between monetary value and HZ vaccine attributes to help price the vaccination programs. Additionally, it is novel that we focused on adults over 25 years instead of 50 years, which largely expanded the study population and made it possible to generalize the results to younger generation.

### Conclusions

In summary, we used DCE to illustrate that Chinese urban adults made trade-offs between HZ vaccination attributes, and effectiveness was the most important characteristic. The residents had the highest WTP for enhancing the effectiveness of vaccines, demonstrating the significance of promoting vaccine effectiveness. We also presented the probability of choosing different HZ vaccines to give suggestions to policy makers on developing and pricing the HZ vaccination programs.

## Appendix

Multimedia Appendix 1 Full version of the questionnaire.

Multimedia Appendix 2 Subgroup analysis.

Multimedia Appendix 3 Univariate marginal estimates for the predicted probability of participation. The x-axis represents the range of probabilities. The y-axis represents the different attributes and levels. The base case is herpes zoster vaccination at 45% protection rate, protection duration of 2 years, vaccination schedule of 1 dose, 1/100 risk of influenza-like symptoms, 1/100 risk of skin reaction, and domestic place of origin, which is indicated as zero change in the probability of the x-axis.

## Abbreviations

DCE: discrete choice experiment
HZ: herpes zoster
WTP: willingness to pay

## Data Availability

The data sets generated during or analyzed during this study are not publicly available to protect the privacy of the participants’ data but are available from the corresponding author on reasonable request.
