# Supplementary material for: Preferences and Willingness to Pay for Herpes Zoster Vaccination Among Chinese Adults: Discrete Choice Experiment
Source: JMIR Public Health Surveill. 2024 Aug 9;10:e51242. doi: 10.2196/51242 (PMC11344184; doi:10.2196/51242)
Supplement: Multimedia Appendix 1 [file publichealth_v10i1e51242_app1.docx]

Preferences for Herpes Zoster Vaccination Survey

Thank you for agreeing to take part in this survey.

Assuming that you have two options for herpes zoster vaccine that differ in price, effectiveness, protection duration, adverse effects, etc., please choose the vaccination option you prefer based on your personal preference among the pairs of options below. When you make a choice, please assume that all conditions are the same for vaccine 1 and vaccine 2 except for the factors listed.

Notes: There are four versions of the DCE questions, each with eight questions, and each respondent is randomly assigned a version of the questions.

Version 1

**Choice Set 1**

|  | Vaccine 1 | Vaccine 2 |
| --- | --- | --- |
| Effectiveness (%) | 90 | 45 |
| Protection duration (years) | 2 | 5 |
| Number of vaccine doses | 2 doses (2 months apart) | 1 dose |
| Probability of influenza-like symptoms | 1/100 | 5/100 |
| Probability of skin reaction | 20/100 | 1/100 |
| Place of origin | Imported | domestic |
| Price (CN ¥) | 0 | 400 |

Which do you prefer: vaccine1; vaccine 2; neither.

**Choice Set 2**

|  | Vaccine 1 | Vaccine 2 |
| --- | --- | --- |
| Effectiveness (%) | 60 | 75 |
| Protection duration (years) | 5 | 10 |
| Number of vaccine doses | 1 dose | 2 doses (2 months apart) |
| Probability of influenza-like symptoms | 5/100 | 10/100 |
| Probability of skin reaction | 5/100 | 10/100 |
| Place of origin | Domestic | imported |
| Price (CN ¥) | 0 | 400 |

Which do you prefer: vaccine1; vaccine 2; neither.

**Choice Set 3**

|  | Vaccine 1 | Vaccine 2 |
| --- | --- | --- |
| Effectiveness (%) | 60 | 75 |
| Protection duration (years) | 10 | 20 |
| Number of vaccine doses | 2 doses (2 months apart) | 1 dose |
| Probability of influenza-like symptoms | 20/100 | 1/100 |
| Probability of skin reaction | 1/100 | 5/100 |
| Place of origin | Imported | domestic |
| Price (CN ¥) | 800 | 1200 |

Which do you prefer: vaccine1; vaccine 2; neither.

**Choice Set 4**

|  | Vaccine 1 | Vaccine 2 |
| --- | --- | --- |
| Effectiveness (%) | 90 | 45 |
| Protection duration (years) | 5 | 10 |
| Number of vaccine doses | 2 doses (2 months apart) | 1 dose |
| Probability of influenza-like symptoms | 20/100 | 1/100 |
| Probability of skin reaction | 5/100 | 10/100 |
| Place of origin | Domestic | imported |
| Price (CN ¥) | 1200 | 0 |

Which do you prefer: vaccine1; vaccine 2; neither.

**Choice Set 5**

|  | Vaccine 1 | Vaccine 2 |
| --- | --- | --- |
| Effectiveness (%) | 60 | 75 |
| Protection duration (years) | 20 | 2 |
| Number of vaccine doses | 2 doses (2 months apart) | 1 dose |
| Probability of influenza-like symptoms | 1/100 | 5/100 |
| Probability of skin reaction | 10/100 | 20/100 |
| Place of origin | Domestic | imported |
| Price (CN ¥) | 400 | 800 |

Which do you prefer: vaccine1; vaccine 2; neither.

**Choice Set 6**

|  | Vaccine 1 | Vaccine 2 |
| --- | --- | --- |
| Effectiveness (%) | 45 | 60 |
| Protection duration (years) | 2 | 5 |
| Number of vaccine doses | 1 dose | 2 doses (2 months apart) |
| Probability of influenza-like symptoms | 1/100 | 5/100 |
| Probability of skin reaction | 1/100 | 5/100 |
| Place of origin | Domestic | imported |
| Price (CN ¥) | 0 | 400 |

Which do you prefer: vaccine1; vaccine 2; neither.

**Choice Set 7**

|  | Vaccine 1 | Vaccine 2 |
| --- | --- | --- |
| Effectiveness (%) | 60 | 75 |
| Protection duration (years) | 20 | 2 |
| Number of vaccine doses | 2 doses (2 months apart) | 1 dose |
| Probability of influenza-like symptoms | 5/100 | 10/100 |
| Probability of skin reaction | 20/100 | 1/100 |
| Place of origin | Domestic | imported |
| Price (CN ¥) | 1200 | 0 |

Which do you prefer: vaccine1; vaccine 2; neither.

**Choice Set 8**

|  | Vaccine 1 | Vaccine 2 |
| --- | --- | --- |
| Effectiveness (%) | 75 | 90 |
| Protection duration (years) | 5 | 10 |
| Number of vaccine doses | 2 doses (2 months apart) | 1 dose |
| Probability of influenza-like symptoms | 1/100 | 5/100 |
| Probability of skin reaction | 20/100 | 1/100 |
| Place of origin | Imported | domestic |
| Price (CN ¥) | 800 | 1200 |

Which do you prefer: vaccine1; vaccine 2; neither.

Version 2

**Choice Set 1**

|  | Vaccine 1 | Vaccine 2 |
| --- | --- | --- |
| Effectiveness (%) | 90 | 45 |
| Protection duration (years) | 5 | 10 |
| Number of vaccine doses | 2 doses (2 months apart) | 1 dose |
| Probability of influenza-like symptoms | 10/100 | 20/100 |
| Probability of skin reaction | 1/100 | 5/100 |
| Place of origin | Domestic | imported |
| Price (CN ¥) | 400 | 800 |

Which do you prefer: vaccine1; vaccine 2; neither.

**Choice Set 2**

|  | Vaccine 1 | Vaccine 2 |
| --- | --- | --- |
| Effectiveness (%) | 75 | 90 |
| Protection duration (years) | 10 | 20 |
| Number of vaccine doses | 1 dose | 2 doses (2 months apart) |
| Probability of influenza-like symptoms | 10/100 | 20/100 |
| Probability of skin reaction | 10/100 | 20/100 |
| Place of origin | Domestic | imported |
| Price (CN ¥) | 0 | 400 |

Which do you prefer: vaccine1; vaccine 2; neither.

**Choice Set 3**

|  | Vaccine 1 | Vaccine 2 |
| --- | --- | --- |
| Effectiveness (%) | 75 | 90 |
| Protection duration (years) | 2 | 5 |
| Number of vaccine doses | 2 doses (2 months apart) | 1 dose |
| Probability of influenza-like symptoms | 20/100 | 1/100 |
| Probability of skin reaction | 5/100 | 10/100 |
| Place of origin | Domestic | imported |
| Price (CN ¥) | 400 | 800 |

Which do you prefer: vaccine1; vaccine 2; neither.

**Choice Set 4**

|  | Vaccine 1 | Vaccine 2 |
| --- | --- | --- |
| Effectiveness (%) | 60 | 75 |
| Protection duration (years) | 2 | 5 |
| Number of vaccine doses | 1 dose | 2 doses (2 months apart) |
| Probability of influenza-like symptoms | 10/100 | 20/100 |
| Probability of skin reaction | 20/100 | 1/100 |
| Place of origin | Imported | domestic |
| Price (CN ¥) | 1200 | 0 |

Which do you prefer: vaccine1; vaccine 2; neither.

**Choice Set 5**

|  | Vaccine 1 | Vaccine 2 |
| --- | --- | --- |
| Effectiveness (%) | 75 | 90 |
| Protection duration (years) | 20 | 2 |
| Number of vaccine doses | 1 dose | 2 doses (2 months apart) |
| Probability of influenza-like symptoms | 5/100 | 10/100 |
| Probability of skin reaction | 1/100 | 5/100 |
| Place of origin | Imported | domestic |
| Price (CN ¥) | 1200 | 0 |

Which do you prefer: vaccine1; vaccine 2; neither.

**Choice Set 6**

|  | Vaccine 1 | Vaccine 2 |
| --- | --- | --- |
| Effectiveness (%) | 60 | 75 |
| Protection duration (years) | 10 | 20 |
| Number of vaccine doses | 2 doses (2 months apart) | 1 dose |
| Probability of influenza-like symptoms | 10/100 | 20/100 |
| Probability of skin reaction | 5/100 | 10/100 |
| Place of origin | Imported | domestic |
| Price (CN ¥) | 0 | 400 |

Which do you prefer: vaccine1; vaccine 2; neither.

**Choice Set 7**

|  | Vaccine 1 | Vaccine 2 |
| --- | --- | --- |
| Effectiveness (%) | 90 | 45 |
| Protection duration (years) | 20 | 2 |
| Number of vaccine doses | 1 dose | 2 doses (2 months apart) |
| Probability of influenza-like symptoms | 10/100 | 20/100 |
| Probability of skin reaction | 10/100 | 20/100 |
| Place of origin | Domestic | imported |
| Price (CN ¥) | 800 | 1200 |

Which do you prefer: vaccine1; vaccine 2; neither.

**Choice Set 8**

|  | Vaccine 1 | Vaccine 2 |
| --- | --- | --- |
| Effectiveness (%) | 90 | 45 |
| Protection duration (years) | 10 | 20 |
| Number of vaccine doses | 1 dose | 2 doses (2 months apart) |
| Probability of influenza-like symptoms | 1/100 | 5/100 |
| Probability of skin reaction | 5/100 | 10/100 |
| Place of origin | Imported | domestic |
| Price (CN ¥) | 1200 | 0 |

Which do you prefer: vaccine1; vaccine 2; neither.

Version 3

**Choice Set 1**

|  | Vaccine 1 | Vaccine 2 |
| --- | --- | --- |
| Effectiveness (%) | 45 | 60 |
| Protection duration (years) | 10 | 20 |
| Number of vaccine doses | 2 doses (2 months apart) | 1 dose |
| Probability of influenza-like symptoms | 5/100 | 10/100 |
| Probability of skin reaction | 20/100 | 1/100 |
| Place of origin | Domestic | imported |
| Price (CN ¥) | 400 | 800 |

Which do you prefer: vaccine1; vaccine 2; neither.

**Choice Set 2**

|  | Vaccine 1 | Vaccine 2 |
| --- | --- | --- |
| Effectiveness (%) | 45 | 60 |
| Protection duration (years) | 10 | 20 |
| Number of vaccine doses | 2 doses (2 months apart) | 1 dose |
| Probability of influenza-like symptoms | 1/100 | 5/100 |
| Probability of skin reaction | 10/100 | 20/100 |
| Place of origin | Domestic | imported |
| Price (CN ¥) | 1200 | 0 |

Which do you prefer: vaccine1; vaccine 2; neither.

**Choice Set 3**

|  | Vaccine 1 | Vaccine 2 |
| --- | --- | --- |
| Effectiveness (%) | 75 | 90 |
| Protection duration (years) | 20 | 2 |
| Number of vaccine doses | 1 dose | 2 doses (2 months apart) |
| Probability of influenza-like symptoms | 1/100 | 5/100 |
| Probability of skin reaction | 5/100 | 10/100 |
| Place of origin | Imported | domestic |
| Price (CN ¥) | 400 | 800 |

Which do you prefer: vaccine1; vaccine 2; neither.

**Choice Set 4**

|  | Vaccine 1 | Vaccine 2 |
| --- | --- | --- |
| Effectiveness (%) | 45 | 60 |
| Protection duration (years) | 20 | 2 |
| Number of vaccine doses | 2 doses (2 months apart) | 1 dose |
| Probability of influenza-like symptoms | 20/100 | 1/100 |
| Probability of skin reaction | 1/100 | 5/100 |
| Place of origin | Imported | domestic |
| Price (CN ¥) | 0 | 400 |

Which do you prefer: vaccine1; vaccine 2; neither.

**Choice Set 5**

|  | Vaccine 1 | Vaccine 2 |
| --- | --- | --- |
| Effectiveness (%) | 75 | 90 |
| Protection duration (years) | 5 | 10 |
| Number of vaccine doses | 2 doses (2 months apart) | 1 dose |
| Probability of influenza-like symptoms | 5/100 | 10/100 |
| Probability of skin reaction | 10/100 | 20/100 |
| Place of origin | Imported | domestic |
| Price (CN ¥) | 0 | 400 |

Which do you prefer: vaccine1; vaccine 2; neither.

**Choice Set 6**

|  | Vaccine 1 | Vaccine 2 |
| --- | --- | --- |
| Effectiveness (%) | 60 | 75 |
| Protection duration (years) | 5 | 10 |
| Number of vaccine doses | 1 dose | 2 doses (2 months apart) |
| Probability of influenza-like symptoms | 1/100 | 5/100 |
| Probability of skin reaction | 1/100 | 5/100 |
| Place of origin | Domestic | imported |
| Price (CN ¥) | 800 | 1200 |

Which do you prefer: vaccine1; vaccine 2; neither.

**Choice Set 7**

|  | Vaccine 1 | Vaccine 2 |
| --- | --- | --- |
| Effectiveness (%) | 90 | 45 |
| Protection duration (years) | 2 | 5 |
| Number of vaccine doses | 2 doses (2 months apart) | 1 dose |
| Probability of influenza-like symptoms | 5/100 | 10/100 |
| Probability of skin reaction | 10/100 | 20/100 |
| Place of origin | Imported | domestic |
| Price (CN ¥) | 800 | 1200 |

Which do you prefer: vaccine1; vaccine 2; neither.

**Choice Set 8**

|  | Vaccine 1 | Vaccine 2 |
| --- | --- | --- |
| Effectiveness (%) | 75 | 90 |
| Protection duration (years) | 2 | 5 |
| Number of vaccine doses | 2 doses (2 months apart) | 1 dose |
| Probability of influenza-like symptoms | 10/100 | 20/100 |
| Probability of skin reaction | 1/100 | 5/100 |
| Place of origin | Domestic | imported |
| Price (CN ¥) | 1200 | 0 |

Which do you prefer: vaccine1; vaccine 2; neither.

Version 4

**Choice Set 1**

|  | Vaccine 1 | Vaccine 2 |
| --- | --- | --- |
| Effectiveness (%) | 45 | 60 |
| Protection duration (years) | 2 | 5 |
| Number of vaccine doses | 1 dose | 2 doses (2 months apart) |
| Probability of influenza-like symptoms | 5/100 | 10/100 |
| Probability of skin reaction | 5/100 | 10/100 |
| Place of origin | Domestic | imported |
| Price (CN ¥) | 800 | 1200 |

Which do you prefer: vaccine1; vaccine 2; neither.

**Choice Set 2**

|  | Vaccine 1 | Vaccine 2 |
| --- | --- | --- |
| Effectiveness (%) | 90 | 45 |
| Protection duration (years) | 10 | 20 |
| Number of vaccine doses | 1 dose | 2 doses (2 months apart) |
| Probability of influenza-like symptoms | 5/100 | 10/100 |
| Probability of skin reaction | 1/100 | 5/100 |
| Place of origin | Imported | domestic |
| Price (CN ¥) | 400 | 800 |

Which do you prefer: vaccine1; vaccine 2; neither.

**Choice Set 3**

|  | Vaccine 1 | Vaccine 2 |
| --- | --- | --- |
| Effectiveness (%) | 75 | 90 |
| Protection duration (years) | 10 | 20 |
| Number of vaccine doses | 1 dose | 2 doses (2 months apart) |
| Probability of influenza-like symptoms | 20/100 | 1/100 |
| Probability of skin reaction | 20/100 | 1/100 |
| Place of origin | Domestic | imported |
| Price (CN ¥) | 800 | 1200 |

Which do you prefer: vaccine1; vaccine 2; neither.

**Choice Set 4**

|  | Vaccine 1 | Vaccine 2 |
| --- | --- | --- |
| Effectiveness (%) | 45 | 60 |
| Protection duration (years) | 5 | 10 |
| Number of vaccine doses | 1 dose | 2 doses (2 months apart) |
| Probability of influenza-like symptoms | 20/100 | 1/100 |
| Probability of skin reaction | 10/100 | 20/100 |
| Place of origin | Imported | domestic |
| Price (CN ¥) | 1200 | 0 |

Which do you prefer: vaccine1; vaccine 2; neither.

**Choice Set 5**

|  | Vaccine 1 | Vaccine 2 |
| --- | --- | --- |
| Effectiveness (%) | 45 | 60 |
| Protection duration (years) | 5 | 10 |
| Number of vaccine doses | 1 dose | 2 doses (2 months apart) |
| Probability of influenza-like symptoms | 10/100 | 20/100 |
| Probability of skin reaction | 20/100 | 1/100 |
| Place of origin | Imported | domestic |
| Price (CN ¥) | 400 | 800 |

Which do you prefer: vaccine1; vaccine 2; neither.

**Choice Set 6**

|  | Vaccine 1 | Vaccine 2 |
| --- | --- | --- |
| Effectiveness (%) | 60 | 75 |
| Protection duration (years) | 2 | 5 |
| Number of vaccine doses | 1 dose | 2 doses (2 months apart) |
| Probability of influenza-like symptoms | 20/100 | 1/100 |
| Probability of skin reaction | 10/100 | 20/100 |
| Place of origin | Imported | domestic |
| Price (CN ¥) | 400 | 800 |

Which do you prefer: vaccine1; vaccine 2; neither.

**Choice Set 7**

|  | Vaccine 1 | Vaccine 2 |
| --- | --- | --- |
| Effectiveness (%) | 90 | 45 |
| Protection duration (years) | 20 | 2 |
| Number of vaccine doses | 1 dose | 2 doses (2 months apart) |
| Probability of influenza-like symptoms | 20/100 | 1/100 |
| Probability of skin reaction | 20/100 | 1/100 |
| Place of origin | Domestic | imported |
| Price (CN ¥) | 0 | 400 |

Which do you prefer: vaccine1; vaccine 2; neither.

**Choice Set 8**

|  | Vaccine 1 | Vaccine 2 |
| --- | --- | --- |
| Effectiveness (%) | 45 | 60 |
| Protection duration (years) | 20 | 2 |
| Number of vaccine doses | 2 doses (2 months apart) | 1 dose |
| Probability of influenza-like symptoms | 10/100 | 20/100 |
| Probability of skin reaction | 5/100 | 10/100 |
| Place of origin | Imported | domestic |
| Price (CN ¥) | 800 | 1200 |

Which do you prefer: vaccine1; vaccine 2; neither.
