# Supplementary material for: Preferences and Willingness to Pay for Herpes Zoster Vaccination Among Chinese Adults: Discrete Choice Experiment
Source: JMIR Public Health Surveill. 2024 Aug 9;10:e51242. doi: 10.2196/51242 (PMC11344184; doi:10.2196/51242)
Supplement: Multimedia Appendix 2 [file publichealth_v10i1e51242_app2.docx]

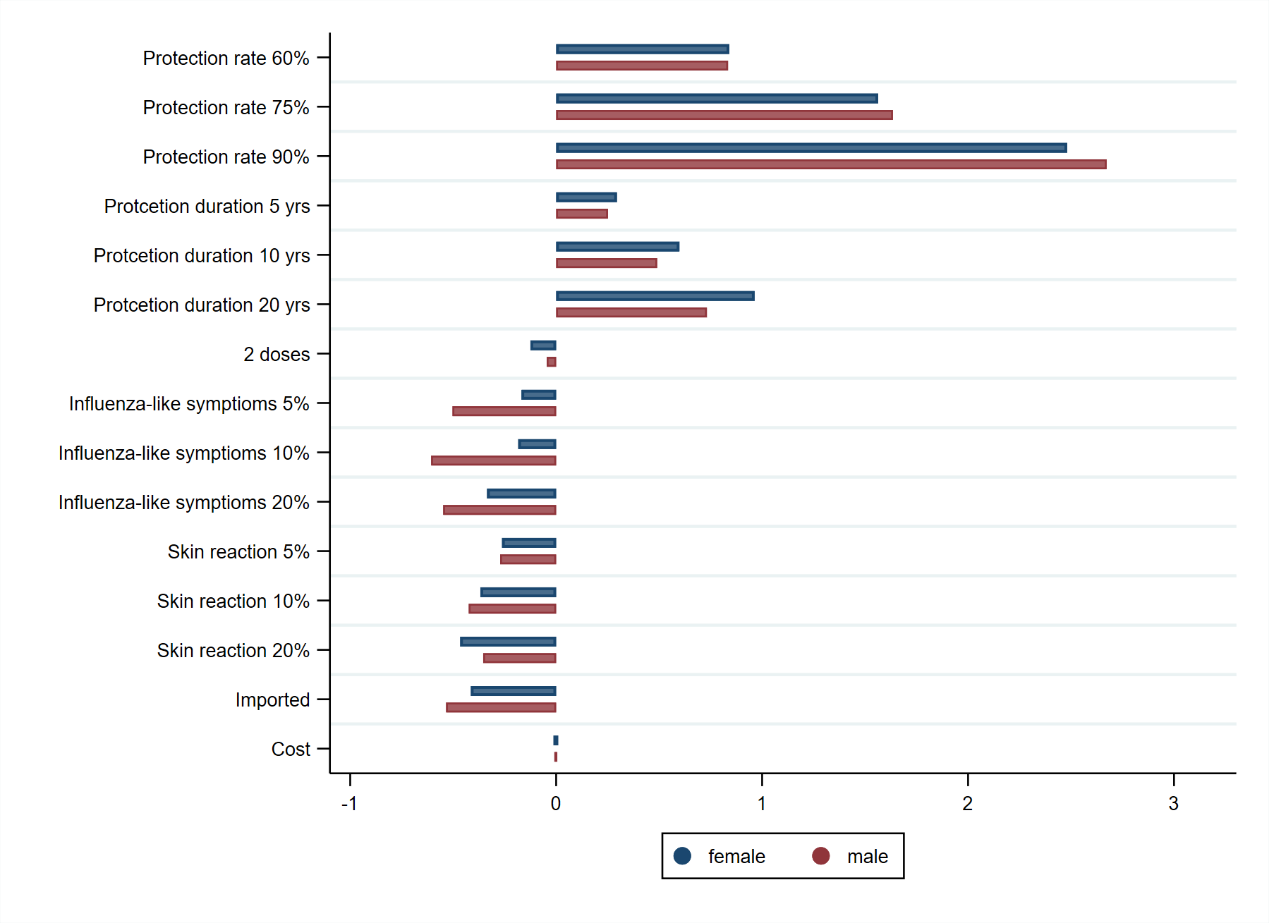


Figure S1 Preferences of attributes of HZ vaccines by gender


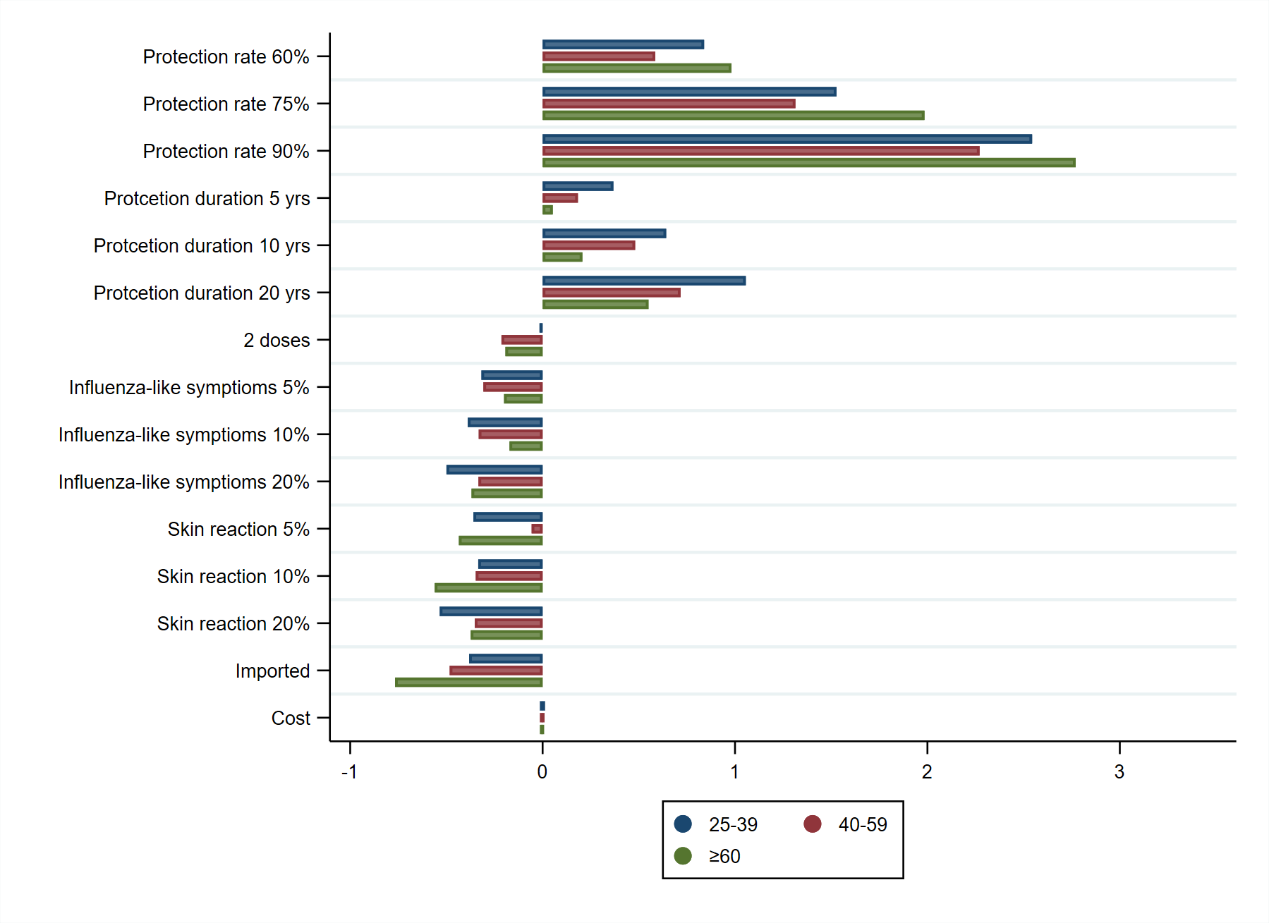


Figure S2 Preferences of attributes of HZ vaccines by age


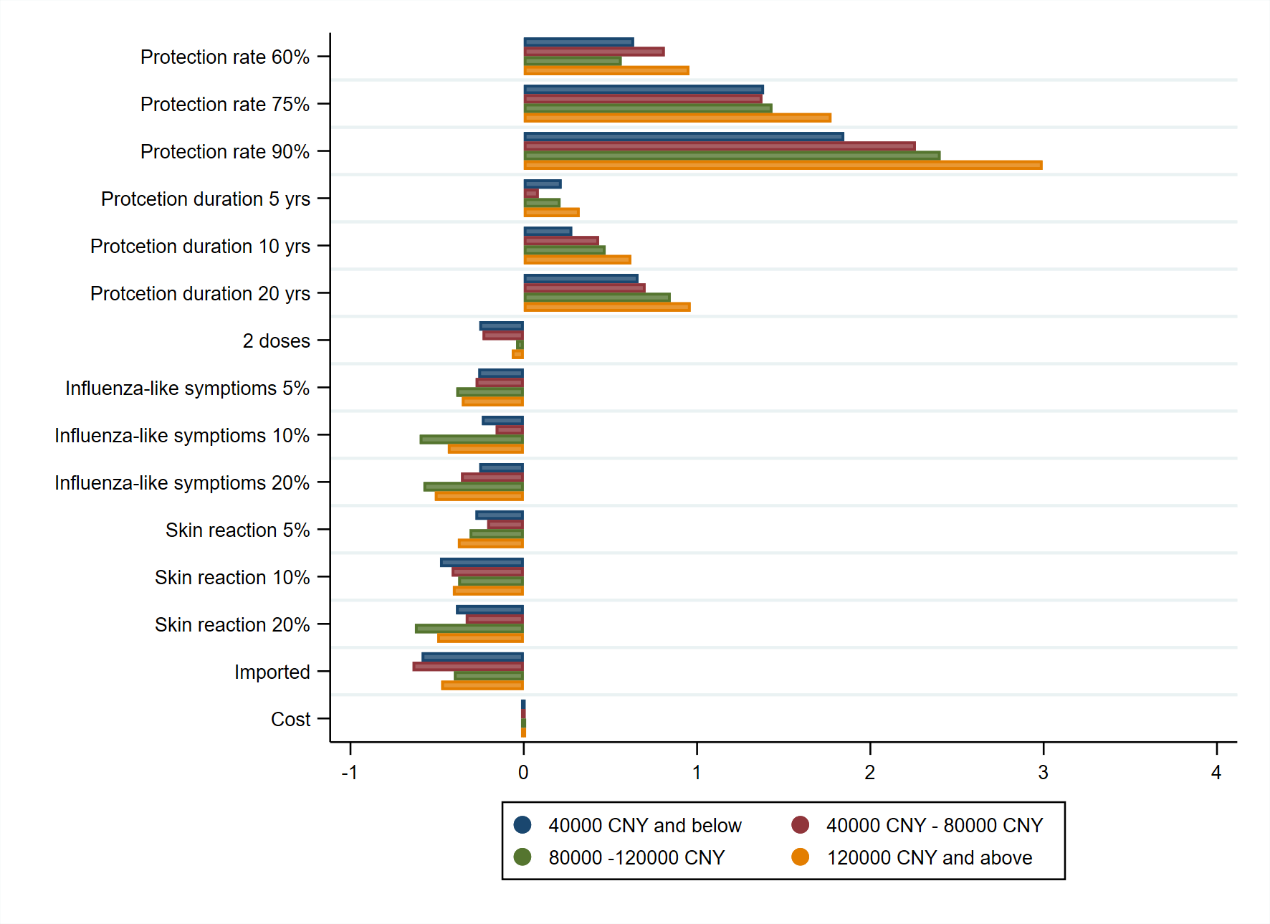


Figure S3 Preferences of attributes of HZ vaccines by annual net household income in 2021
