# Supplementary figures and images for: Preferences and Willingness to Pay for Herpes Zoster Vaccination Among Chinese Adults: Discrete Choice Experiment
Source: JMIR Public Health Surveill. 2024 Aug 9;10:e51242. doi: 10.2196/51242 (PMC11344184; doi:10.2196/51242)

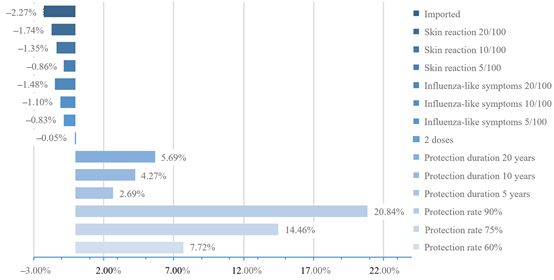

Supplement: Multimedia Appendix 3 [file publichealth_v10i1e51242_app3.png]
